# Hip fractures in Chinese TikTok (Douyin) short videos: an analysis of information quality, content and user comment attitudes

**DOI:** 10.3389/fpubh.2025.1563188

**Published:** 2025-04-24

**Authors:** Zhuoxin Li, Yashi Lin, Kairou Zhang, Ran Li, Mei Ju, Yanhua Chen, Jing Fu, Ruiyu Huang, Ling Zhu, Junjun Sun, Yanxia Guo, Min Gao, Yue Hu, Gang Liu, Baolu Zhang

**Affiliations:** ^1^Department of Nursing, The Affiliated Hospital, Southwest Medical University, Luzhou, China; ^2^School of Nursing, Southwest Medical University, Luzhou, China; ^3^School of Continuing Education, Guiyang Healthcare Vocational University, Guiyang, China; ^4^Department of Chinese Medicine, Sichuan Provincial People's Hospital, University of Electronic Science and Technology of China, Chengdu, China; ^5^School of Nursing, Xinxiang Medical University, Xinxiang, China; ^6^Faculty of Nursing and Midwifery, Jiangsu College of Nursing, Huaian, China; ^7^Department of Orthopedics and Center for Orthopedic Diseases Research, Affiliated Traditional Chinese Medicine Hospital of Southwest Medical University, Luzhou, China

**Keywords:** hip fractures, Douyin, information quality, short video, user comment attitudes

## Abstract

**Background:**

Hip fracture presents a major healthcare challenge globally. While numerous Douyin videos address hip fracture, their information quality and factors affecting user comment attitudes remain uncertain.

**Objective:**

This study aims to analyze the content, information quality, and user comment attitudes of videos depicting hip fractures on Chinese TikTok (Douyin).

**Methods:**

The search term “hip fracture” was used on Douyin, which resulted in 170 samples being included. Video information quality was assessed using the GQS and PEMAT scales. Video content was analyzed using DivoMiner. User comments were extracted using Gooseeker, and user comment attitudes were interpreted as positive, neutral, or negative using the Weiciyun website. Data analysis was performed using IBM SPSS version 29.0, including non-parametric tests for continuous variables and chi-square tests for categorical variables. The identified factors were then included in a multivariate logistic regression analysis to examine their impact on user comment attitudes.

**Results:**

Health professionals were the primary source of videos (136/138, 98.6%). The overall information quality of the videos was moderate (median 3, IQR 2.00–4.00). Douyin videos were relatively high in understandability (median 72.70%, IQR 63.60–81.80%) but low in actionability (median 33.33%, IQR 0–66.67%). Most videos focused on treatment (139/170, 81.8%). Regarding user comment attitudes, the majority of videos were received with positive comments (113/170, 66.5%), followed by negative comments (39/170, 22.9%) and neutral comments (18/170, 10.6%). The multivariate logistic regression analysis revealed three factors influencing positive attitudes: the GQS score (OR 13.824, 95% CI 6.033–31.676), understandability (OR 2.281, 95% CI 1.542–5.163) and not mentioning risk factors in videos (OR 0.291, 95%CI 0.091–0.931).

**Conclusion:**

The majority of hip fracture videos on Douyin were created by health professionals and had intermediate information quality, with user comment attitudes remaining positive. However, these videos often lacked actionability and had insufficient mention of prevention and rehabilitation content. Videos with higher information quality that addressed hip fracture risk factors received more positive user comments. This study suggests that publishers of hip fracture-related videos should improve actionability while simultaneously paying attention to both prevention and rehabilitation content to enhance the educational value of these videos.

## 1 Introduction

Hip fracture refers to the fracture of the proximal leg bone (1), which has become a major healthcare problem and is characterized by high incidence (2), high mortality (3), and substantial healthcare burden (4). Global hip fracture incidence was estimated to exceed 14 million cases in 2019 (5). This figure is projected to double by 2050 (6). The one-year mortality rate following hip fracture is 3–4 times higher than that of the general population (7).

Although short videos on platforms such as Douyin have inherent limitations in comprehensive health education due to time constraints, there is research evidence supporting the effectiveness of short videos for specific health education objectives (8). Studies have shown that short video health education interventions can significantly improve health knowledge levels and patient satisfaction, particularly in orthopedic care settings (9). The advantages of short video formats are reflected in their high engagement and viewing completion rates—research has found that health education videos under 100 s have a viewing retention rate approaching 100%, with patients typically not interrupting viewing (10). For health issues such as hip fractures that require preventive knowledge and treatment understanding, these easily accessible short videos provide an important entry point for obtaining basic information, especially considering research showing that older adults are increasingly using social media to obtain health guidance (11). Further studies indicate that even brief videos can effectively deliver key health information, thereby enhancing public awareness and prevention consciousness regarding specific diseases (12).

In recent years, health information dissemination has undergone significant changes, with video platforms emerging as new channels for public access to healthcare knowledge (13, 14). Among various social media platforms, Douyin (the Chinese version of TikTok) has proven to be an effective platform for health-related information dissemination (15). As of January 2024, Douyin reportedly had 1.62 billion users (16). Douyin and TikTok operate under ByteDance, sharing similar technological infrastructure and features, with Douyin serving the domestic Chinese market while TikTok targets international audiences. Data indicates that 200 million users engage with health-related content on the platform daily (17). While social media platforms have facilitated patient access to health information, the proliferation of unverified health content poses potential risks to patient safety and well-being (18).

Given the critical importance of information quality for patient safety, researchers have begun evaluating the information quality of health-related videos across various medical conditions on Douyin. For instance, lung cancer-related videos on Douyin demonstrate intermediate information quality (19). In contrast, videos about cosmetic surgery and oral diabetes on Douyin exhibit high information quality (20, 21). However, the information quality of hip fracture health education videos on Douyin remains unclear.

Beyond examining information quality, understanding user comments on health education videos is equally important. Through textual content, emoticons, and other forms of expression, user comments enable individuals to comprehensively share their views and attitudes (22). While likes count serves as a basic indicator of user preferences on social platforms (23, 24), user comment attitudes represent deeper insights into their content preferences and perceptions. Existing studies have primarily focused on like counts as metrics (25–27). However, analyzing user comments offers richer qualitative insights into audience perceptions and content preferences. Therefore, this study will evaluate both the information quality and content of relevant videos, while analyzing user comment attitudes and their influencing factors.

## 2 Materials and methods

### 2.1 Search strategy and data collection

**Figure 1:**
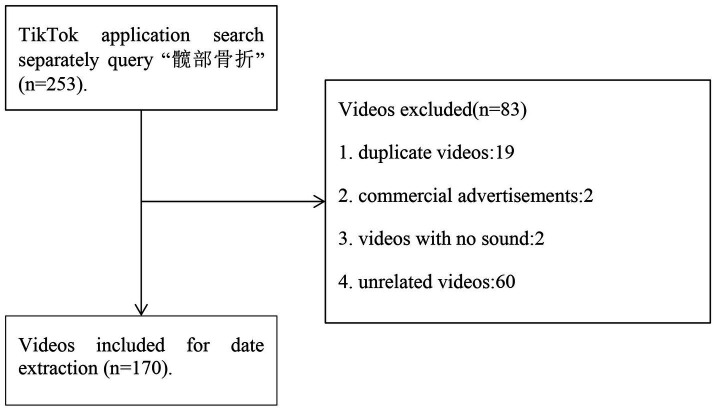
Flowchart for screening videos related to hip fracture on Douyin platform.

This study conducted a basic search on the Douyin platform using the Chinese term “髋部骨折 (which means “hip fracture”) on July 13, 2023. Researcher A collected all the video links (a total of 253) based on the platform’s comprehensive ranking. The comprehensive ranking approach mitigates potential biases toward merely popular or recent content. While TikTok’s algorithm customizes content recommendations based on individual preferences, this integrated ranking methodology aligns well with users’ authentic behavioral patterns when seeking health-related information. This sampling method has been widely used in other Douyin video analysis studies (18, 23). To maintain consistency in the available video pool, we searched within a single day due to the platform’s daily content updates. All video links, including those directly related to hip fractures, were stored in Microsoft Excel. Exclusion criteria included duplicate videos, commercial advertisements, videos without sound, and unrelated content. Following this screening, 170 videos were retained for further data extraction and analysis (Figure 1).

The screening process was conducted independently by two researchers (Researcher A and Researcher B). Specifically, an initial screening was performed by examining video titles and descriptions to identify and exclude obvious advertisements and irrelevant content. Subsequently, the researchers manually reviewed each preliminarily screened video to confirm whether it substantially addressed hip fractures. Silent videos were identified by checking for audio output during playback; duplicate videos were identified by comparing video IDs and content; commercial advertisements were determined based on whether the video content contained product promotions, pricing information, and purchase links; irrelevant content referred to videos that contained the keyword “hip fracture” but primarily discussed other topics. In cases of disagreement between the two researchers, consensus was reached through discussion or by consulting a third researcher (Researcher C) to resolve discrepancies.

This study did not use account verification status (i.e., the so-called “Blue V” and “Yellow V” certified accounts- where Blue V represents verified individual accounts of public figures or experts, and Yellow V represents verified institutional accounts such as hospitals or media organizations) as a criterion for video screening. Instead, it included all videos that met the thematic relevance requirements, regardless of whether their publishers were verified by the platform. This decision was based on the following considerations: First, we aimed to comprehensively evaluate hip fracture-related information available on the Douyin platform; second, non-certified accounts may also provide valuable health information; third, ordinary users would likewise encounter videos published by unverified accounts when searching for related content.

Two researchers (Researcher A and Researcher B) extracted video features (days posted, length, likes, shares, and user comments) and publisher characteristics (sources, publisher gender, department, and professional title) from all filtered video links between July 14 and 18, 2023. This process was mainly done manually, i.e., the researcher visited each video page and the publisher’s official account homepage one by one to record the relevant information. For video characteristics, they were obtained directly by visiting the video page; for publisher characteristics, they were based on self-reported information on their official account homepage. To ensure the accuracy of data extraction, Researcher A and Researcher B cross-validated 10% of the sample data, achieving a Cohen’s Kappa coefficient of *κ* = 0.89, indicating almost perfect inter-rater agreement. For the collection of user comments, this study utilized Gooseeker (28). Gooseeker is a free web data capture tool in China known for its ease of use (29). Unlike Python, Gooseeker includes built-in web scraping features, requiring no manual coding and making it easy to learn. All data were organized in Microsoft Excel.

Regarding the identification of publisher characteristics (such as gender, professional background, and department), we employed the following methods. First, publisher gender was determined through a comprehensive assessment of profile pictures, self-introductions on their account homepages, and their appearance in videos. Next, medical professionals were identified based on professional certification badges on their account homepages, affiliated hospital or medical institution information, displays of professional qualification certificates, and self-descriptions in their account profiles. Then, orthopedic surgeons were more specifically identified according to their explicitly stated departmental affiliations in their profiles (such as “orthopedic surgeon,” “orthopedic director,” etc.), their hospital and department information, and the depth of professional knowledge demonstrated in their video content. Finally, for accounts with incomplete or ambiguous information, researchers implemented a two-step verification process: first, they further examined the account’s historical video content and comment interactions on the Douyin platform; if questions remained, we cross-verified through search engines such as Baidu to find public information about the relevant doctors or medical institutions. This additional verification was primarily used to confirm the authenticity of publishers’ professional backgrounds, especially for accounts claiming to be medical professionals but with insufficient platform information.

### 2.2 Evaluation methodology and procedure

The evaluation of hip fracture-related videos on Douyin was conducted in three areas: information quality, video content, and user comment attitudes. This study selected PEMAT-A/V and GQS as assessment tools rather than DISCERN, primarily based on the alignment between research objectives and tool characteristics. First, PEMAT-A/V is specifically designed for audiovisual materials and can effectively evaluate the understandability and actionability of short videos (30), which is particularly important for assessing the brief yet information-dense content on Douyin. GQS provides a comprehensive assessment framework for information quality, fluency, and usability (31), which is suitable for the multidimensional evaluation needs of hip fracture video content in this study.

In contrast, DISCERN primarily focuses on assessing treatment choice information, with 9 of its 16 questions specifically targeting treatment options (32), while this study addresses not only treatment information but also comprehensive content, including prevention, rehabilitation, and risk factors. Recent systematic reviews (33) indicate that although DISCERN is widely used in health video assessment, its framework, originally designed for written materials, presents applicability challenges when applied to short videos. Recent literature (8, 23) has confirmed that PEMAT-A/V and GQS assessment tools demonstrate good applicability in social media short video platforms, particularly in evaluating the educational value and practicality of content.

First, we used the PEMAT-A/V (Patient Education Materials Assessment Tool for Audiovisual Materials) and the GQS (Global Quality Scale) to evaluate the video information quality. The PEMAT-A/V was used to assess the understandability and actionability of the patient education audiovisual materials. The tool consists of two parts: understandability (questions 1–13) and actionability (questions 14–17). The PEMAT-A/V score above 70% indicates that information is easy to understand or act on, while a score below 70% suggests lower understandability or actionability (30). Details are provided in Supplementary Table 1. The GQS is a 5-point scale used to rate videos based on information quality, flow, and ease of use. A video with a GQS score of 4 or 5 points is regarded as high quality, while a video with a score of 3 points is considered intermediate, and a video with a score of 1 or 2 points is considered low quality (31). Details are provided in Supplementary Table 2. Two independent raters (a clinical physician with 6 years of clinical experience at the attending physician level and Researcher D) independently conducted information quality assessments, strictly following rating procedures to ensure reliability. When disagreements occurred, a clinical orthopedic expert with 10 years of clinical experience and a senior professional title was consulted to reach consensus. Inter-rater reliability was calculated using IBM SPSS Statistics 29.0. For the 17 items in the PEMAT-A/V tool, inter-rater reliability was 0.869, while 0.832 for the GQS tool, reflecting satisfactory consistency between raters.

Second, we analyzed the video content using DivoMiner (34). DivoMiner is a text-mining and automatic content analysis platform supported by machine learning algorithms (35). Based on clinical guidelines for hip fractures and themes identified through researchers’ review of video content, our study categorized the hip fracture video content into eight primary domains: definition, symptoms, causes, treatment, risk factors, prevention, rehabilitation, and complications (36, 37). Each of the eight areas was coded as dichotomous variables (mentioned vs. not mentioned). The detailed codebook is provided in the Supplementary Table 3. The researchers set the codebook in the category management of DivoMiner and imported the textual content of all 170 videos into DivoMiner. The Holsti coefficient between them was calculated using the DivoMiner reliability function, yielding a result of 0.84. It indicates good inter-coder reliability and allows for further analysis. The same two raters analyzed all video content, employing identical disagreement resolution procedures.

Finally, we analyze user comment attitudes by utilizing the Weiciyun platform (38). Weiciyun is a professional and user-friendly web text analysis software. It is easy to use and delivers excellent results (39). Compared to similar tools, Weiciyun offers superior Chinese text processing capabilities. When handling large volumes of comment data, the platform provides an efficient and accurate automated analysis solution.

The classification of each video is based on the predominant attitudes expressed in the comments. The specific steps involved are as follows: (1) importing comments extracted from each video; (2) incorporating custom emotional words and adjusting emoji settings, including terms such as “like,” “heart,” “clap,” and “thank you” to represent positive attitudes, as well as “sobbing,” “cracking up,” and “awkward laughter” for negative attitudes; (3) automatically analyzing the proportions of attitudes (positive, neutral, negative) in each comment; and (4) assigning attitude categories according to the highest percentage identified.

### 2.3 Statistical analysis

Data analysis was conducted using IBM SPSS Statistics 29.0. Variables were categorized as either categorical or continuous. Categorical data were described as frequencies (%), while continuous variables, given their nonnormal distribution, were presented as medians (interquartile range, IQR). Chi-square and Kruskal-Wallis H tests were used to compare categorical and continuous variables, respectively, with statistical significance set at *p* < 0.05. Although the dependent variable was ordinal, the independent variable did not meet the assumption of parallel lines (*p* < 0.001), necessitating a multinomial logistic regression analysis. After collinearity testing (tolerance > 0.1, VIF < 10) and standardization, significant variables were included in the final multinomial logistic regression model to assess their impact on user comment attitudes.

### 2.4 Ethical considerations

This study analyzed public videos about hip fractures and related user comments on TikTok. As the study did not directly involve human participants and only utilized publicly accessible information, ethical review was not required.

## 3 Results

### 3.1 Video characteristics

Table 1 outlines the basic characteristics of videos based on user comment attitudes. This study analyzed 170 videos related to hip fractures. Based on user comment attitudes, the videos were categorized into three groups: Positive, Neutral, and Negative. Most videos received positive comments (113/170, 66.5%), followed by negative (39/170, 22.9%) and neutral (18/170, 10.6%). In terms of publisher characteristics, male publishers significantly dominated the production of hip fracture-related videos, accounting for 94.1% (160/170) of the total sample. A statistically significant difference was observed in gender distribution across different user comment attitudes (*p* = 0.007), with the highest proportion of male publishers found in videos receiving positive user comment attitudes (96.5%, 109/113). Regarding departmental distribution, the majority of the videos were uploaded by health professionals (159/170, 93.5%), with orthopedic surgeons being the primary content publishers (92.9%, 158/170). The distribution of departments demonstrated significant differences across user comment attitude groups (*p* = 0.001), with videos receiving negative user comment attitudes showing the highest proportion of orthopedic surgeons (97.4%, 38/39).

**Table 1:** Basic characteristics of videos based on user comment attitudes.

Variables	Positive (*n* = 113)	Neutral (*n* = 18)	Negative (*n* = 39)	Overall (*n* = 170)	Statistics	*p* value
Source, *n* (%)^a^						
Health professional	108 (95.6)	14 (77.8)	37 (94.9)	159 (93.5)	9.187	0.057
News agencies	2 (1.8)	1 (5.6)	0 (0.0)	3 (1.8)		
Nonprofit organizations	3 (2.6)	3 (16.6)	2 (5.1)	8 (4.7)		
Publisher gender, *n* (%)^a^						
Male	109 (96.5)	14 (77.8)	37 (94.9)	160 (94.1)	9.841	0.007
Female	4 (3.5)	4 (22.2)	2 (5.1)	10 (5.9)		
Department, *n* (%)^a^						
Orthopedics	107 (94.7)	13 (72.7)	38 (97.4)	158 (92.9)	13.506	0.001
Others	6 (5.3)	5 (27.8)	1 (2.6)	12 (7.1)		
Days posted, median (IQR)^b^	217.00 (69.50–500.00)	183.50 (29.50–427.75)	300.00 (66.00–645.00)	235.50 (67.50–513.50)	2.961	0.228
Length (Sec), median (IQR)^b^	67.00 (43.50–97.50)	77.00 (46.25–112.00)	64.00 (41.00–99.00)	66.50 (43.75–98.25)	0.392	0.822
Likes, median (IQR)^b^	215.00 (102.50–530.00)	118.00 (48.25–323.25)	207.00 (74.00–719.00)	205.50 (95.00–608.25)	4.096	0.129
Shares, median (IQR)^b^	28.00 (11.00–110.00)	26.00 (7.50–129.00)	49.00 (18.00–99.00)	33.50 (11.75–109.00)	1.534	0.464
User comment count, median (IQR)^b^	24.00 (9.00–52.50)	8.00 (2.50–40.25)	38.00 (9.00–95.00)	23.00 (8.00–61.25)	6.236	0.044

a *p-values were calculated with the chi-square test.*

b *p-values were calculated with the Kruskal-Wallis H test.*

For all included videos, the median time since upload was 235.50 days (IQR 67.50–513.50). The median number of likes was 215.00 (IQR 95.00–608.25), shares 33.50 (IQR 11.75–109.00), and user comments 23.00 (IQR 8.00–61.25). Further analysis of user comments revealed varying engagement levels across attitude groups, with negative-attitude videos receiving the highest median number of comments (38.00, IQR 9.00–95.00), followed by positive-attitude videos (24.00, IQR 9.00–52.50), while neutral-attitude videos had the lowest median comment count (8.00, IQR 2.50–40.25). This difference was statistically significant (*p* = 0.044) (Table 1).

### 3.2 Information quality

Table 2 shows the information quality of hip fracture videos on Douyin, assessed according to PEMAT-A/V and GQS and categorized by user comment attitudes. The analysis of information quality revealed varying levels across different dimensions. Regarding understandability, the videos demonstrated a relatively high overall score (median 72.70%, IQR 63.60–81.80%), with significant differences observed across user comment attitude groups (*p* = 0.005). Videos receiving positive user comment attitudes exhibited the highest understandability scores (72.70–81.80%). In contrast, the actionability of the videos was notably low (median 33.33%, IQR 0–66.67%). GQS assessment indicated a moderate overall quality (median 3.00, IQR 2.00–4.00), with videos receiving positive user comment attitudes demonstrating higher scores (median 3.00, IQR 3.00–4.00). The results of the Kruskal-Wallis test showed that both GQS (*p* < 0.001) and understandability (*p* < 0.05) differed significantly across user comment attitude groups.

**Table 2:** PEMAT A/V scores and GQS scores of videos by user comment attitudes.

Variables	Positive (*n* = 113)	Neutral (*n* = 18)	Negative (*n* = 39)	Overall (*n* = 170)	Statistics	*p* value
PEMAT-A/V, median (IQR)^a^						
Understandability (%)	72.70 (70.00–81.80)	73.85 (63.60–80.00)	63.60 (54.50–77.80)	72.70 (63.60–81.80)	0.005	0.005
Actionability (%)	33.33 (0–66.67)	33.33 (33.33–66.67)	33.33 (0–66.67)	33.33 (0–66.67)	0.081	0.081
GQS, median (IQR)^a^	3.00 (3.00–4.00)	2.00 (1.00–3.00) ^b^	2.00 (1.00–2.00) ^b^	3.00 (2.00–4.00)	68.389	<0.001

a *p values were calculated with the Kruskal-Wallis H test.*

b Significant differences from the positive attitude group: neutral (*p* < 0.001), negative (*p* < 0.001).

### 3.3 Video content

Table 3 describes the video content classified according to the user comment attitudes. The hip fracture-related videos evaluated in this study covered eight topics: definitions, symptoms, causes, treatments, risk factors, prevention methods, rehabilitation, and complications. Treatment was the most common topic (139/170, 81.8%). Conversely, Rehabilitation and Prevention methods were mentioned the least with 27 (15.9%) and 34 (20.0%), respectively. Notably, videos with positive user comment attitudes focused more on Risk Factors (51.3% vs. 28.2%) compared to those with negative attitudes, showing a statistically significant difference in this aspect (*p* < 0.05) (Table 3).

**Table 3:** Video content of videos by user comment attitudes.

Variables	Positive (*n* = 113)	Neutral (*n* = 18)	Negative (*n* = 39)	Overall (*n* = 170)	Statistics	*p* value
Definitions, *n* (%)^a^	46 (40.7)	7 (38.9)	16 (41.0)	69 (40.6)	0.025	0.987
Symptoms^a^	45 (39.8)	9 (50.0)	19 (48.7)	73 (42.9)	1.346	0.510
Causes^a^	38 (33.6)	6 (33.3)	12 (30.8)	56 (32.9)	0.109	0.947
Treatments^a^	90 (79.6)	14 (77.8)	35 (89.7)	139 (81.8)	2.197	0.333
Risk factors^a^	58 (51.3)	9 (50.0)	11 (28.2)	78 (45.9)	6.380	0.041
Prevention^a^	26 (23.0)	3 (16.7)	5 (12.8)	34 (20.0)	2.021	0.364
Rehabilitation^a^	17 (15.0)	4 (22.2)	6 (15.4)	27 (15.9)	0.608	0.738
Complications^a^	29 (25.7)	9 (50.0)	14 (35.9)	52 (30.6)	5.003	0.082

a *p values were calculated with the chi-square test.*

### 3.4 Multivariate logistics regression analysis

Table 4 describes the results of the multivariate logistic regression analysis. Based on the test results, six independent variables were included in the final multivariate logistic regression analysis. Four variables (GQS, PEMAT-A/V understandability, Risk Factors, and department) significantly impacted user comment attitudes, while publisher gender and user comment count had no effect. The analysis revealed that GQS (*p* < 0.001), PEMAT-A/V understandability (*p* < 0.001), and Risk Factors (*p* = 0.038) had a significant impact on user comment attitudes.

**Table 4:** Multivariate logistics regression analysis of influencing factors of different user comment attitudes.

Dependent variables	Independent variables	*b*	SE(b)	Wald *x*^2^	*P* value	OR	95%CI
Positive^a^	GQS	2.626	0.423	38.540	<0.001	13.824	6.033–31.676
	PEMAT-A/V understandability	1.037	0.308	11.320	<0.001	2.821	1.542–5.163
	Risk factors (not mentioned)	−1.235	0.594	4.325	0.038	0.291	0.091–0.931
Neutral^b^	GQS	−1.913	0.417	21.006	<0.001	0.148	0.065–0.335
	Department (orthopedics)	−2.756	1.001	7.580	0.006	0.064	0.009–0.452
Negative^b^	GQS	−2.626	0.423	38.540	<0.001	0.072	0.032–0.166
	PEMAT-A/V understandability	−1.037	0.308	11.320	<0.001	0.354	0.194–0.649
	Risk factors (not mentioned)	1.235	0.594	4.325	0.038	3.437	1.074–11.004

a Utilize the negative group as a reference.

b Utilize the positive group as a reference.

On one hand, comparing videos with positive user comment attitudes to those with negative attitudes, the odds ratio (OR) for GQS in positive-attitude videos was 13.824 (95% CI 6.033–31.676), indicating a significantly higher ratio than in negative-attitude videos, where the OR was 0.072 (95% CI 0.032–0.166). Additionally, for videos with positive user comment attitudes, the OR for PEMAT-A/V understandability was 2.821 (95% CI 1.542–5.163), while the OR for negative-attitude videos was 0.354 (95% CI 0.194–0.649). On the other hand, the OR of not mentioning risk factors in videos with positive user comment attitudes was 0.291 (95% CI 0.091–0.931), and the OR of not mentioning risk factors in videos with positive user comment attitudes was 3.437 (95% CI 1.074–11.004) (Table 4).

## 4 Discussion

### 4.1 Principal findings

This study evaluated the information quality, content characteristics, and user comment attitudes of hip fracture-related videos on TikTok. The findings indicate that most videos were posted by health professionals. The overall information quality was moderate and generally received positive user comment attitudes. Notably, videos with higher information quality and those addressing risk factors for hip fractures tended to receive more positive user comment attitudes. However, the actionability of these videos still needs improvement. Additionally, the video content primarily focused on hip fracture treatment, with limited coverage of prevention and rehabilitation.

Regarding information quality, this study found that most videos were posted by health professionals. This may be because hip fracture, as a complex orthopedic condition, involves medical knowledge across multiple professional domains including diagnosis, surgical planning, and postoperative rehabilitation, making it challenging for non-medical professionals to accurately grasp and communicate related content. The moderate overall information quality aligns with findings from studies on chronic pain management and probiotics on social media (40, 41), but differs from research on diabetes content on TikTok and osteoporosis content on YouTube (18, 42) due to different assessment tools - they used DISCERN (32) focusing on treatment aspects, while we employed GQS (43) for overall quality measurement. Based on these findings, publishers should enhance video information quality while maintaining professionalism.

Additionally, this study found that these videos demonstrated appropriate comprehensibility, enabling users from diverse backgrounds and health literacy levels to understand hip fracture-related knowledge. Similar comprehensibility features were also observed in video studies related to oral epithelial dysplasia and basal cell carcinoma (44, 45), reflecting that short-form health education videos generally emphasize audience reception. However, the actionability of videos is an aspect worth discussing. Previous studies on health-related social media content have also identified actionability as a common area requiring attention (46–49). This phenomenon may stem from publishers focusing on knowledge dissemination while giving relatively less attention to translating knowledge into practical action. Although videos enable users from different backgrounds to understand content through accessible expression, understanding alone may not fully meet the needs. Users face certain challenges in making specific health decisions or taking corresponding actions based on video content, which may affect the videos’ practical effectiveness in health education. We suggest that publishers focus on enhancing video actionability while maintaining appropriate comprehensibility.

User comment attitudes showed general positivity toward hip fracture-related videos, particularly demonstrating more favorable reactions to content with higher information quality, highlighting the importance of enhancing healthcare video information quality. Furthermore, additional research found that videos addressing hip fracture risk factors also received positive comment attitudes from users, likely because understanding disease risk factors helps viewers identify their own risks and heighten disease awareness, thus encouraging them to engage more actively in discussions and share their perspectives (50). We recommend that future video production should not only focus on improving information quality but also incorporate content about hip fracture risk factors to better meet users’ viewing needs and expectations.

Regarding video content, this study found that more than half of the videos focused on hip fracture treatment, with minimal attention to rehabilitation and prevention. Prevention encompasses a range of measures designed to minimize or eliminate diseases, addressing their root causes, complications, and recurrence through risk factor reduction, disease progression control, and management of existing condition impacts (51). Prevention before hip fractures occurs has significant impacts on reducing morbidity and mortality rates, while also lowering healthcare costs and raising public health awareness (52–54). Additionally, rehabilitation can improve patients’ daily living abilities (55, 56) and enhance their quality of life. However, hip fracture patients typically lack sufficient understanding of fractures and rehabilitation (57), highlighting the importance of prevention and rehabilitation. This study recommends increasing content related to hip fracture prevention and rehabilitation in video production. Specifically, content publishers should consider: (1) Creating short videos (30–60 s) specifically focused on prevention strategies or rehabilitation exercises, which aligns with the effective video duration observed in our study; (2) Enhancing the actionability of videos, as our research indicates that while existing videos have high comprehensibility, they generally demonstrate low actionability—this can be achieved through visual demonstrations accompanied by clear step-by-step guidance, enabling viewers not only to understand the content but also to take concrete actions based on it; (3) Incorporating content related to risk factors, as our analysis reveals that videos mentioning risk factors received significantly more positive comments; (4) Producing more rehabilitation-focused content by orthopedic specialists, as our research indicates they are the primary trusted publishers, yet rarely address rehabilitation content.

## 5 Limitations and future directions

This study has several limitations. First, our sample was confined to data collected on a single day (July 13, 2023), which may not account for potential fluctuations caused by Douyin’s dynamic algorithm updates. Additionally, we did not conduct a comparison of data collected across multiple days to verify the stability of our findings. Future studies should consider collecting samples across multiple days and comparing data from different time points to ensure the robustness of results against algorithmic fluctuations inherent to social media platforms. Second, our sample was confined to Douyin, and findings may differ from platforms in other languages. Third, while user-friendly, the Weiciyun platform has limitations in analyzing contextual meaning, sarcasm, and cultural references in comments; future research should employ more sophisticated natural language processing techniques, such as deep learning models to capture semantic subtleties that simpler text mining approaches might miss. Additionally, the interaction effects between GQS and other video quality metrics may have affected our estimation of GQS influence, with hierarchical modeling approaches needed in future research. Despite these limitations, this study provides the first systematic investigation into how information quality and content of hip fracture videos on Douyin influence user comment attitudes.

## 6 Conclusion

This study analyzed information quality, content, and user comment attitudes about hip fracture on the Douyin platform. The study reveals that hip fracture videos on Douyin demonstrate intermediate overall information quality, with a primary emphasis on treatment content. However, these videos rarely address prevention and rehabilitation aspects, while also presenting limited actionability. In addition, users’ attitudes toward these videos were mostly positive and preferred videos with high information quality and mention of risk factors. We recommend that future content publishers enhance the information quality of their videos, while incorporating additional content on hip fracture rehabilitation, prevention, and associated risk factors. Content publishers should focus on developing concise videos that effectively demonstrate prevention strategies and rehabilitation exercises, while ensuring orthopedic specialists address key risk factors to maximize educational impact.

## Data Availability

The data analyzed in this study was obtained from Douyin (Chinese TikTok) platform, subject to the platform’s terms of use. The extracted data and analysis results are available from the corresponding authors upon reasonable request.
