# Supplementary material for: Hip fractures in Chinese TikTok (Douyin) short videos: an analysis of information quality, content and user comment attitudes
Source: Front Public Health. 2025 Apr 24;13:1563188. doi: 10.3389/fpubh.2025.1563188 (PMC12058786; doi:10.3389/fpubh.2025.1563188)
Supplement: Supplementary file 1 [file Table_1.docx]

Supplementary Material

**Supplementary Table 1**: Patient Education Materials Assessment Tool for Audiovisual (PEMAT-A/V).

| Item | Description |
| --- | --- |
| Section 1: Understandability |  |
| 1 | The material makes its purpose completely evident (*P* and A/V) |
| 3 | The material uses common, everyday language (*P* and A/V) |
| 4 | Medical terms are used only to familiarize the audience with the terms. When used, medical terms are defined (*P* and A/V) |
| 5 | The material uses the active voice (*P* and A/V) |
| 8 | The material breaks or "chunks" information into short sections (*P* and A/V) |
| 9 | The material's sections have informative headers (*P* and A/V) |
| 10 | The material presents information in a logical sequence (*P* and A/V) |
| 11 | The material provides a summary (*P* and A/V) |
| 12 | The material uses visual cues to draw attention to key points (*P* and A/V) |
| 13 | Text on the screen is easy to read (A/V) |
| 14 | The material allows the user to hear the words clearly (A/V) |
| 18 | The material allows the user to hear the words clearly (A/V)) |
| 19 | The material uses simple tables with short and clear row and column headings (*P* and A/V) |
| Section 2: Actionability |  |
| 20 | The material clearly identifies at least one action the user can take (*P* and A/V) |
| 21 | The material addresses the user directly when describing actions (*P* and A/V) |
| 22 | The material breaks down any action into manageable, explicit steps (*P* and A/V) |
| 25 | The material explains how to use the charts, graphs, tables, or diagrams to take actions (P and A/V) |

Note: Each item is rated as 'Yes' (1), 'No' (0), or 'Not Applicable (NA)'. A 'Yes' is given when 80-100% of the material meets the criterion. For audiovisual material under 1 minute or with ≤6 slides/screenshots, items are marked 'NA'. The final score is: (Total score / Maximum possible score) × 100.

**Supplementary Table 2**: Global Quality Score (GQS) (Scoring ranges from 1 to 5).

| Score | Definition |
| --- | --- |
| 1 | Poor quality, poor flow of the video, most information missing, not at all useful for patients |
| 2 | Generally poor quality and poor flow, some information listed but many important topics missing, of very limited use to patients |
| 3 | Moderate quality, some important information is adequately discussed |
| 4 | Good quality good flow, most relevant information is covered, useful for patients |
| 5 | Excellent quality and flow, very useful for patients |

Note: The GQS evaluates videos using a five-point scoring system from poor (1) to excellent (5) quality.

**Supplementary Table 3**: Coding Book for Hip Fracture Videos.

| Code | Definition | Examples |
| --- | --- | --- |
| Definitions | Videos explain what a hip fracture is and identify the common sites where hip fractures occur. | -A fracture caused by trauma that results in a  break in the femoral neck.  -This primarily refers to fractures below the  femoral neck's base and above the lesser  trochanter.  -It is also known as an intertrochanteric fracture. |
| Symptoms | Videos primarily discuss the common clinical symptoms observed in patients with hip fractures. | -This condition can lead to prolonged bed rest.  -Patients may experience hip pain and an inability to stand.  -There may be active bleeding, limb shortening, and a limp. |
| Causes | Videos discuss the primary causes leading to hip fractures. | -Many elderly individuals may suffer hip fractures due to slipping or accidental falls while using the bathroom.  -Car accidents can also result in hip fractures.  -Hip dysplasia can contribute to the occurrence of hip fractures. |
| Treatments | Videos present the clinical approaches for treating hip fractures, the outcomes of these treatments, and the factors influencing the choice of treatment methods. | -Clinical treatment is primarily divided into surgical and conservative approaches. Surgical therapies include hip arthroplasty and total hip replacement.  -Conservative treatment may lead to various complications, whereas patients undergoing surgical treatment can typically mobilize quickly and have a better prognosis.  -Surgery should be performed as soon as possible in patients who are physically able and can tolerate the procedure. For individuals over 65 with femoral neck fractures, joint replacement surgery is generally recommended. |
| Risk factors | Videos discuss potential risk factors that may lead to hip fractures. | -Hip fractures primarily occur in the elderly, who are more prone to osteoporosis, making them susceptible to fractures.  -Hip fractures represent the most severe complication of osteoporosis.  -The elderly are at increased risk of femoral neck fractures due to decreased muscle strength and coordination, making them more vulnerable to trauma.  -The incidence is higher in women than in men.  -The supplementation of calcium and vitamin D is crucial. |
| Prevention | Videos explain common methods for preventing hip fractures in the elderly. | -Efforts should be made to prevent falls in the elderly by eliminating potential risk factors, such as avoiding overly soft sofas, installing non-slip mats in the bathroom, and placing grab bars near toilets for support.  -Calcium and vitamin D supplements should be taken to improve bone density, with regular bone density testing recommended.  -Engaging in moderate physical activity, using anti-osteoporosis medications.  -Adjusting diet while correcting unhealthy lifestyle habits are also important. |
| Rehabilitation | Videos guide rehabilitation exercises following hip fracture surgery. | -Postoperative patients can engage in early mobilization and functional exercises to expedite their return to pre-injury life.  -Strength training focused on the legs is recommended, and squatting is one of the most convenient exercises for leg conditioning in daily life. |
| Complications | Videos discuss other diseases that may arise in hip fracture patients due to prolonged bed rest. | -Prolonged bed rest in hip fracture patients can lead to pulmonary infections, such as hypostatic pneumonia.  -Deep vein thrombosis (DVT) in the lower limbs may dislodge and cause embolism in vital organs, resulting in severe complications or even death.  -Patients may develop urinary retention, which can lead to urinary tract infections. |
